# Clinical comparison between thoracoscopic and thoracotomy repair of Gross type C esophageal atresia

**DOI:** 10.1186/s12893-021-01360-7

**Published:** 2021-11-22

**Authors:** Shen Yang, Peize Wang, Zhi Yang, Siqi Li, Junmin Liao, Kaiyun Hua, Yanan Zhang, Yong Zhao, Yichao Gu, Shuangshuang Li, Yongwei Chen, Jinshi Huang

**Affiliations:** 1 Department of Neonatal Surgery, Beijing Children’s Hospital, Capital Medical University, National Center for Children’s Health, 56 Nanlishi Road, Beijing, 100045 China; 2 Department of Neonatal Surgery, The Affiliated Children’s Hospital of Nanchang University, Nanchang, 330006 China

**Keywords:** Esophageal atresia, Thoracoscopic, Thoracotomy, Comparison, Complications, Outcome

## Abstract

**Background:**

To compare the clinical outcomes between thoracoscopic approach and thoracotomy surgery in patients with Gross type C Esophageal atresia (EA) and tracheoesophageal fistula (TEF).

**Methods:**

Patients with Gross type C EA/TEF who underwent surgery from January 2007 to January 2020 at Beijing Children’s Hospital were retrospectively analyzed. The patients were divided into two groups according to surgical approaches. The perioperative factors and postoperative complications were compared among the two groups.

**Results:**

One hundred and ninety patients (132 boys and 58 girls) with a median birth weight of 2975 (2600, 3200) g were included. The primary operations were performed via thoracoscopic (n = 62) and thoracotomy (n = 128) approach. After comparison of clinical characteristics between the two groups, we found that there were statistically significant differences in associated anomalies, method of fistula closure, duration of mechanical ventilation after surgery, feeding option before discharge, management of pneumothorax, and prognosis (all *P* < 0.05). To a certain extent, thoracoscopic surgery reduced the incidence of anastomotic leakage and increased the incidence of anastomotic stricture in this study. However, there were no statistically significant differences between the two groups in terms of operative time, postoperative pneumothorax, anastomotic leakage, anastomotic stricture, and recurrent tracheoesophageal fistula (all *P* > 0.05).

**Conclusions:**

Thoracoscopy surgery for Gross type C EA/TEF is a safe and effective, minimally invasive technique with comparable operative time and incidence of postoperative complications.

**Supplementary Information:**

The online version contains supplementary material available at 10.1186/s12893-021-01360-7.

## Introduction

Esophageal atresia (EA) and tracheoesophageal fistula (TEF) is one of the most common congenital malformations of the esophagus, with an incidence of 1/2500–1/4500 [1]. Since Dr Cameron Height performed the first successful primary repair of a neonate with EA/TEF in 1941, advances in surgical technique and neonatal care have steadily improved survival rates of babies within the EA/TEF spectrum [2]. The survival rate of Gross type C EA/TEF without severe malformation reported in the relevant literature is higher than 90% [3].

With the development of minimally invasive technology, several studies have reported superior visualization of the anatomy, reduced pain, a lower degree of skeletal deformities and minimal scarring after thoracoscopic EA/TEF repair, which has the benefits of being minimally invasive and is as effective as open surgery in terms of operating time, blood loss, postoperative ventilation time and postoperative leaks and strictures [4, 5]. However, the procedure is technically demanding due to the restricted working space of the neonatal thorax combined with inherent difficulties of using thoracoscopic instruments to perform an esophageal anastomosis under tension, and therefore has not replaced thoracotomy.

The aim of this study was to compare the clinical outcomes between thoracoscopic approach and thoracotomy surgery in patients with Gross type C EA/TEF.

## Materials and methods

### Patients

One hundred and ninety patients with Gross type C EA/TEF who underwent surgery from January 2007 to January 2020 at Beijing Children’s Hospital were included in this study. Their demographic information, preoperative assessments, operative details, and complications were extracted from the electronic medical records and retrospectively analyzed. Specific outcomes of interest included the short-term postoperative complications that occurred during the initial hospital stay, as well as any evidence of long-term sequelae noted during the follow-up clinic visits. Z-score medians and ranges were calculated for all four growth indicators (height for age, weight for height, weight for age, and BMI for age) using the World Health Organization’s Anthro Software (Version 3.2.2, WHO, Geneva, Switzerland). The patients were retrospectively divided into two groups according to whether they received thoracoscopic or thoracotomy surgery. The perioperative factors and postoperative complications were compared among the two groups. This retrospective study was approved by the Medical Ethics Committee of the Beijing Children’s Hospital (2019-k-333), and the patient informed consent requirements were waived.

### Statistical analysis

We analyzed all the data on SPSS 25.0. Continuous variables were presented as the mean with standard deviation or median and interquartile range if the normality hypothesis test rejected the null hypothesis of normal distribution. Categorical variables were reported as counts and percentages. Two independent samples t-tests and *χ*^2^ tests were used to compare characteristics between the thoracoscopic and thoracotomy groups. *P* < 0.05 was considered statistically significant.

## Results

### Patient characteristics

As shown in Table 1, in this study, 190 patients were included in the analysis (132 boys and 58 girls). These patients had a median birth weight of 2975 (2600, 3200) g and 22 (11.58%) patients were preterm. The median age at time of operation of all patients was 4 (3, 7) days. One hundred fifty-eight patients were found to have other congenital diseases, including nonsyndromic anomalies (n = 143), VACTERL syndrome (n = 14), and syndromic diagnosis (n = 1).

**Table 1:** Clinical characteristics

Variables		Thoracoscopy (n = 62)	Thoracotomy (n = 128)	Results	*p*
Gender (n, %)	Boy	44 (70.97)	88 (68.75)	0.097	0.756
	Girl	18 (29.03)	40 (31.25)		
Age at first surgery (median, days)		4 (3, 7)	4 (3, 7)	-0.048	0.962
Gestational age (n, %)	Preterm	6 (9.68)	16 (12.50)	0.325	0.569
	Term/overdue	56 (90.32)	112 (87.50)		
Birth weight (median, g)		2900 (2673, 3200)	3000 (2500, 3260)	-0.245	0.806
Distance (median, cm)		2.00 (1.00, 2.50)	1.50 (0.70, 2.25)	-1.932	0.053
Associated anomalies (n, %)	Yes	57 (91.94)	101 (78.91)	5.063	0.024
	No	5 (8.06)	27 (21.09)		
Mechanical ventilation before surgery (n, %)	Yes	4 (6.45)	9 (7.03)	0.000	1.000
	No	58 (93.55)	119 (92.97)		

All patients underwent surgery after the diagnosis of EA/TEF. Primary operations were performed via thoracotomy (n = 128) and thoracoscopic (n = 62) approaches. Six patients underwent converted to an open thoracotomy surgery because of decreased oxygen saturation (n = 3) and high esophageal anastomosis tension (n = 3).

As shown in Table 2, after a median follow-up of 68 (22, 117) months, 140 patients survived, 11 died (including 6 patients whose parents refused and abandoned treatment after surgical repair of EA/TEF, 2 patients who died of complications after surgical repair of EA/TEF, 1 who died of severe multiple abnormalities, 1 who died of perforation after dilation procedure, and 1 who died due to an unknown reason), and 39 lost to follow-up. Gastroesophageal reflux occurred in 26 patients (including 2 patients who underwent Nissen fundoplication), history of respiratory problems (such as persistent coughing, recurrent pneumonia, and asthma) was noted in 25 patients. Two patients presented with thoracic skeletal anomalies, and both of them were treated by thoracotomy. Additional file 1: Table S1 shows the results of growth evaluation during the follow-up by recording the height and weight centiles of all survivors.

**Table 2:** Comparison between thoracoscopy and thoracotomy groups

Variables		Thoracoscopy (n = 62)	Thoracotomy (n = 128)	Results	*p*
Operative time (median, minutes)		125.5 (90.0, 206.3)	130.0 (110.0, 150.0)	− 0.222	0.825
Method for fistula closure (n, %)	Transfixing suture	7 (11.48)	116 (94.31)	128.294	< 0.001
	Ham-lock clip	22 (36.07)	0 (0)		
	Ligation	32 (52.46)	7 (5.69)		
Duration of mechanical ventilation after surgery (median, hours)		141.0 (117.5, 170.5)	93.0 (28.5, 212.5)	− 2.309	0.021
Duration of intensive care unit stay after surgery (median, days)		11 (8, 19)	13 (6, 23)	− 0.327	0.744
Parenteral nutrition treatment (median, days)		18 (13, 23)	15 (10, 22)	− 1.921	0.055
Days until starting liquid diet (median, days)		13 (9, 16)	12 (9, 19)	− 0.068	0.945
Feeding option before discharge (n, %)	Full oral	45 (72.58)	78 (60.94)	9.366	0.009
	Tube feeding	10 (16.13)	11 (8.59)		
	Total parenteral nutrition	7 (11.29)	39 (30.47)		
Total of duration of hospitalization (median, days)		25 (20, 34)	23 (18, 34)	− 0.606	0.544
Pneumothorax (n, %)	Yes	25 (40.98)	56 (44.09)	0.163	0.687
	No	36 (59.02)	71 (55.91)		
Management of pneumothorax (n, %)	Observation	10 (40.00)	14 (25.00)	7.806	0.020
	Only Thoracentesis	8 (32.00)	8 (14.29)		
	Closed thoracic drainage (with/without thoracentesis)	7 (28.00)	34 (60.71)		
Anastomotic leakage (n, %)	Yes	19 (30.65)	55 (42.97)	2.668	0.102
	No	43 (69.35)	73 (57.03)		
Anastomotic stricture (n, %)	Yes	25 (40.32)	23 (27.71)	2.549	0.110
	No	37 (59.68)	60 (72.29)		
Recurrent tracheoesophageal fistula (n, %)	Yes	7 (11.29)	6 (7.32)	0.679	0.410
	No	55 (88.71)	76 (92.68)		
Prognosis (n, %)	Survival	54 (87.10)	86 (67.19)	8.640	0.011
	Death	2 (3.23)	9 (7.03)		
	Lost-to follow up	6 (9.68)	33 (25.78)		
History of gastroesophageal reflux (n, %)	Yes	16 (29.63)	10 (15.63)	–	–
	No	38 (70.37)	54 (84.38)		
History of respiratory problems (n, %)	Yes	5 (10.20)	20 (30.77)	–	–
	No	44 (89.80)	45 (69.23)		

### Comparison between the thoracoscopy and thoracotomy groups

Tables 1 and 2 show the differences of clinical characteristics between the two groups. There were statistically significant differences for associated anomalies, method of fistula closure, duration of mechanical ventilation after surgery, feeding option before discharge, management of pneumothorax, and prognosis (all *P* < 0.05). To a certain extent, thoracoscopic surgery reduced the incidence of anastomotic leakage and increased the incidence of anastomotic stricture in this study. However, there were no statistically significant differences between the two groups in terms of operative time, duration of intensive care unit after surgery, total of duration of hospitalization, postoperative pneumothorax, anastomotic leakage, anastomotic stricture, and recurrent tracheoesophageal fistula (all *P* > 0.05).

## Discussion

Since the first report of thoracoscopic repair of EA/TEF in 1999, thoracoscopic surgery for EA/TEF has gradually been proven to be safe and feasible [6]. Compared with thoracotomy, thoracoscopic surgery can allow easier esophageal dissection with excellent visualization and can avoid the thoracotomy incision which is associated with more pain and skeletal deformities. Several reports have assessed the safety and effectiveness of thoracoscopic surgery. Elbarbary et al. showed comparable outcomes between the thoracoscopic and the open method for short-gap type C EA/TEF [7]. Yamoto et al. demonstrated that the thoracoscopic approach was favorable and safe for EA/TEF repair in carefully selected patients [8]. A meta-analysis carried out in 2016 concluded that there were no significant differences between thoracoscopy and thoracotomy groups with respect to anastomotic leaks and strictures. In addition, patients who had received thoracoscopic surgery were extubated faster, started oral feeding earlier, and stayed in the hospital for a shorter period of time. However, it was noted that their operative time was longer [9].

The reported rate of conversion from thoracoscopy to thoracotomy surgery in literature ranges from 4 to 44% [10]. In the present study, 6 patients (9.68%) were converted to thoracotomy surgery due to decreased oxygen saturation or high esophageal anastomosis tension. All conversions took place before 2016 as shown in Additional file 2: Table S2. With more experience, none of the thoracoscopic procedures had to be converted to open surgery anymore. In addition, compared to before 2016, the duration of operation time decreased significantly, and there was a remarkable reduction in postoperative leakage from 58 to 19% (Additional file 2: Table S2). We believe that increased surgical expertise and the technical adjustments led to this reduction in postoperative leakage. As this study has demonstrated, there is a clearly learning curve, but upon overcoming the learning curve and mastering the intracorporeal knotting, the operative time of thoracoscopy was comparable to thoracotomy.

In the present study, we found that the duration of mechanical ventilation after surgery of thoracoscopy was longer than that of thoracotomy. The main reason was the improvement of perioperative management system which enabled more patients to be admitted to the intensive care unit for mechanical ventilation after thoracoscopy surgery. Mechanical ventilation helps avoiding the impact of autonomous respiration and swallowing on the esophageal anastomosis. However, we found no significant differences in thoracoscopy versus thoracotomy in regard to duration of stay in the intensive care unit after surgery, length of time before starting liquid diet and hospitalization length.

Wu et al. analyzed the outcomes that are universally considered to be indicators of the effectiveness of the thoracoscopic EA/TEF repair, namely, conversion to thoracotomy surgery, the rates of complications, anastomotic leaks and strictures. In their meta-analysis, they demonstrated statistically insignificant differences in all the parameters considered [11]. In the present study, there were no significant differences in the rates of pneumothorax, anastomotic leakage, anastomotic stricture, and recurrent tracheoesophageal fistula between the two groups. To a certain extent, thoracoscopic surgery reduced the incidence of anastomotic leakage and increased the incidence of anastomotic stricture in this study. However, 74 patients with anastomotic leakage were all healed after fasting, parenteral nutrition support, and insertion of a gastric tube. It has been reported that endoscopic balloon dilatation is an effective method for the treatment of anastomotic stricture [12, 13]. In our study, patients received balloon dilatation due to esophageal stricture, and the median number of dilations for both groups was 6. As for the higher incidence rate of anastomotic stricture in the thoracoscopic group, we analyzed that it might be related to the more stitches and the smaller proximal pouch opening during the thoracoscopic anastomosis. At present, in our clinical work, we are still improving the thoracoscopic technique to further improve the safety of surgery and reduce postoperative complications.

There were several limitations in our study. On the one hand, a high rate of loss to follow-up (39/190, 20.53%) has an impact on the outcome. We found that 38 patients (38/39, 97.44%) were lost-to follow up before standardized EA/TEF treatment and management procedures (complete follow-up procedures were established in 2016 in our hospital). In order to create a more effective postoperative long-term care and treatment, it is fundamental to ensure a standardized, multidisciplinary follow-up that must continue until adulthood. On the other hand, the criteria for decisions related to surgical procedures and perioperative and postoperative management were impacted by the experience and improvement of the treatment plan, as well as the establishment of clinical teams and changes in practice over time. Thoracotomy accounted for the vast majority before 2014 in our hospital. With the promotion and maturity of endoscopic technology, the number of thoracoscopic surgeries has begun to dominate and there are no absolute contraindications. Thus, a prospective randomized controlled trial is needed to explore the differences in clinical outcomes between thoracoscopy and thoracotomy surgery for Gross type C EA/TEF in the future.

## Conclusion

In conclusion, the findings of this study show that thoracoscopy surgery of Gross type C EA/TEF is a safe and effective, minimally invasive technique with comparable outcomes and operative time. For experienced surgeons, thoracoscopy can serve as a first-line technique for EA/TEF repair.

## Supplementary Information

**Additional file 1: Table S1.** Growth evaluation of Gross type C EA/TEF patients.

**Additional file 2: Table S2.** Clinical comparison of thoracoscopic surgery before and after 2016.

## Data Availability

All data generated or analyzed during this study are included in this published article [and its additional information files].
