# Supplementary material for: Clinical comparison between thoracoscopic and thoracotomy repair of Gross type C esophageal atresia
Source: BMC Surg. 2021 Nov 22;21:403. doi: 10.1186/s12893-021-01360-7 (PMC8607600; doi:10.1186/s12893-021-01360-7)
Supplement: Supplementary file 1 — Additional file 1: Table S1. Growth evaluation of Gross type C EA/TEF patients. [file 12893_2021_1360_MOESM1_ESM.docx]

Table S1. Growth evaluation of Gross type C EA/TEF patients

| Variables | | Z-scores | Pathological Z-scores (< -2SD) (n, %) |
| --- | --- | --- | --- |
| < 5 years  (n = 47) | WAZ | -0.15 (-0.86, 0.53) | 5 (10.64) |
|  | HAZ | -0.66 (-1.64, 1.19) | 10 (21.28) |
|  | WHZ | 0.11 (-0.73, 1.02) | 3 (6.38) |
| 5-10 years  (n = 41) | WAZ | -0.11 (-1.29, 1.10) | 5 (12.20) |
|  | HAZ | -0.12 (-0.88, 1.04) | 2 (4.88) |
|  | BAZ | -0.79 (-1.94, 0.89) | 10 (24.39) |
| > 10 years  (n = 22) | HAZ | 0.36 (-0.07, 1.02) | 1 (4.55) |
|  | BAZ | -0.51 (-2.13, 0.37) | 6 (27.27) |

BAZ: BMI for age; HAZ: height for age; WAZ: weight for age; WHZ: weight for height.
