# Supplementary material for: Clinical comparison between thoracoscopic and thoracotomy repair of Gross type C esophageal atresia
Source: BMC Surg. 2021 Nov 22;21:403. doi: 10.1186/s12893-021-01360-7 (PMC8607600; doi:10.1186/s12893-021-01360-7)
Supplement: Supplementary file 2 — Additional file 2: Table S2. Clinical comparison of thoracoscopic surgery before and after 2016. [file 12893_2021_1360_MOESM2_ESM.docx]

Table S2. Clinical comparison of thoracoscopic surgery before and after 2016

| Variables | | Before 2016 (n = 19) | After 2016 (n =43) | Results | *p* |
| --- | --- | --- | --- | --- | --- |
| Converted to thoracotomy（n, %） | | 6 (31.58) | 0 (0) | - | *-* |
| Operative time (median, min) |  | 220.0 (180.0, 255.0) | 101.0 (86.0, 135.0) | -5.010 | < 0.001 |
| Pneumothorax (n, %) | Yes | 9 (47.37) | 16 (37.21) | 0.565 | 0.452 |
|  | No | 10 (52.63) | 27 (62.79) |  |  |
| Anastomotic leakage (n, %) | Yes | 11 (57.89) | 8 (18.60) | 9.571 | 0.002 |
|  | No | 8 (42.11) | 35 (81.40) |  |  |
| Anastomotic stricture (n, %) | Yes | 2 (10.53) | 24 (55.81) | 11.099 | 0.001 |
|  | No | 17 (89.47) | 19 (44.19) |  |  |
| Recurrent tracheoesophageal fistula (n, %) | Yes | 3 (15.79) | 4 (9.30) | 0.554 | 0.457 |
|  | No | 16 (84.21) | 39 (90.70) |  |  |
| Prognosis (n, %) | Survival | 13 (68.42) | 41 (95.35) | 15.560 | < 0.001 |
|  | Death | 0 | 2 (4.65) |  |  |
|  | Lost-to follow up | 6 (31.58) | 0 |  |  |
